# Description of *Komagataeibacter* *melaceti* sp. nov. and *Komagataeibacter melomenusus* sp. nov. Isolated from Apple Cider Vinegar

**DOI:** 10.3390/microorganisms8081178

**Published:** 2020-08-03

**Authors:** Leon Marič, Ilse Cleenwerck, Tomaž Accetto, Peter Vandamme, Janja Trček

**Affiliations:** 1 Department of Biology, Faculty of Natural Sciences and Mathematics, University of Maribor, SI-2000 Maribor, Slovenia; leeon.maric@gmail.com; 2 BCCM/LMG Bacteria Collection, Laboratory of Microbiology, Ghent University, Faculty of Sciences, B-9000 Ghent, Belgium; Ilse.Cleenwerck@UGent.be (I.C.); peter.vandamme@ugent.be (P.V.); 3 Animal Science Department, Biotechnical Faculty, University of Ljubljana, SI-1230 Domžale, Slovenia; Tomaz.Accetto@bf.uni-lj.si; 4 Faculty of Chemistry and Chemical Engineering, University of Maribor, SI-2000 Maribor, Slovenia

**Keywords:** Acetic acid bacteria, *Acetobacteraceae*, *Komagataeibacter*, *Komagataeibacter melaceti*, *Komagataeibacter melomenusus*, vinegar

## Abstract

Two novel strains AV382 and AV436 were isolated from a submerged industrial bioreactor for production of apple cider vinegar in Kopivnik (Slovenia). Both strains showed very high (≥98.2%) 16S rRNA gene sequence similarities with *Komagataeibacter* species, but lower 16S–23S rRNA gene internal transcribed spacer (ITS). The highest similarity of the 16S–23S rRNA gene ITS of AV382 was to *Komagataeibacter kakiaceti* LMG 26206^T^ (91.6%), of AV436 to *Komagataeibacter xylinus* LMG 1515^T^ (93.9%). The analysis of genome sequences confirmed that AV382 is the most closely related to *K. kakiaceti* (ANIb 88.2%) and AV436 to *K. xylinus* (ANIb 91.6%). Genome to genome distance calculations exhibit for both strains ≤47.3% similarity to all type strains of the genus *Komagataeibacter*. The strain AV382 can be differentiated from its closest relatives *K. kakiaceti* and *Komagataeibacter saccharivorans* by its ability to form 2-keto and 5-keto-D-gluconic acids from glucose, incapability to grow in the presence of 30% glucose, formation of C_19:0_ cyclo *ω*8c fatty acid and tolerance of up to 5% acetic acid in the presence of ethanol. The strain AV436 can be differentiated from its closest relatives *K. xylinus*, *Komagataeibacter sucrofermentans,* and *Komagataeibacter nataicola* by its ability to form 5-keto-D-gluconic acid, growth on 1-propanol, efficient synthesis of cellulose, and tolerance to up to 5% acetic acid in the presence ethanol. The major fatty acid of both strains is C_18:1_ *ω*7c. Based on a combination of phenotypic, chemotaxonomic and phylogenetic features, the strains AV382^T^ and AV436^T^ represent novel species of the genus *Komagataeibacter*, for which the names *Komagataeibacter* *melaceti* sp. nov. and *Komagataeibacter melomenusus* are proposed, respectively. The type strain of *Komagataeibacter melaceti* is AV382^T^ (= ZIM B1054^T^ = LMG 31303^T^ = CCM 8958^T^) and of *Komagataeibacter melomenusus* AV436^T^ (= ZIM B1056^T^ = LMG 31304^T^ = CCM 8959^T^).

## 1. Introduction

The genus *Komagataeibacter* taxonomically belongs to the family *Acetobacteraceae* in the class *α-Proteobacteria*. Additionally, it represents one of the 19 genera of a group which is classified as acetic acid bacteria [1,2]. The genus was described in 2013 by redefinition of the genus *Gluconacetobacter* which resulted in reclassification of the species from the *Gluconacetobacter xylinus* clade to a new genus *Komagataeibacter* [3,4]. The main phenotypic features which differentiate the genus *Komagataeibacter* from *Gluconacetobacter* are absence of flagellation, absence of water-soluble brown pigment production when grown on glucose-yeast-calcium-carbonate medium, and absence of 2,5-diketo-D-glucose production from glucose [3,5]. As accessed on 28 May 2020 from the website of List of Prokaryotic names with Standing in Nomenclature (LPSN) [6], the genus *Komagataeibacter* contains 17 species which were isolated from various sugar and alcohol containing substrates, such as vinegars, fruits, kombucha, coconut milk, nata de coco, and beet juice [7,8,9,10,11,12,13]. The genus *Komagataeibacter* gained immense interest among researchers due to the massive and efficient production of nanocellulose by members of the species *Komagataeibacter xylinus*, *Komagataeibacter medellinensis*, *Komagataeibacter hansenii*, *Komagataeibacter nataicola*, *Komagataeibacter oboediens*, *Komagataeibacter rhaeticus*, *Komagataeibacter saccharivorans* and *Komagataeibacter pomaceti* [14]. The nanocellulose can be *in situ* or *ex situ* modified to different biomedical products [15].

The phylogeny of acetic acid bacteria has been well-established on a comparison of 16S rRNA gene sequences, but especially for the genus *Komagataeibacter* other taxonomic approaches have also been used, such as multilocus sequence analysis of housekeeping genes [16], (GTG)_5_-PCR [17] and MALDI-TOF MS [18]. Additionally, sequencing of 16S-23S rRNA gene internal transcribed spacer was established as a reliable approach for species identification of acetic acid bacteria [19]. This intergenic region contains two highly conserved tRNA but also segments that remained evolutionary enough conserved to identify species [20]. It has been successfully used for tentative identification of some novel species of acetic acid bacteria [12,13].

In 2017, an industrial apple cider vinegar producing submerged bioreactor was sampled for acetic acid bacteria from different production batches aiming to establish a large pool of diverse strains for various biotechnological applications. Before starting screening of strains for specific applications, all strains were tentatively taxonomically described by phenotypic and molecular methods. In this way, strains AV382 and AV436, isolated from different batches, have been identified as potentially novel species. Both strains have been, according to their phenotypic characteristics and taxonomic gene markers, affiliated with the genus *Komagataeibacter*, but differed in their 16S-23S rRNA gene ITS regions significantly from each other and from the type strains of this genus. Therefore, we proposed that both strains represent novel *Komagataeibacter* species. Further genome sequence analysis and a detailed phenotypic analysis were performed to determine the taxonomic position of both strains.

## 2. Materials and Methods

### 2.1. Isolation and Cultivation of Strains

During industrial apple cider vinegar production in Kopivnik, a settlement in the north-eastern part of Slovenia, the vinegar probes were systematically sampled from submerged bioprocesses onto RAE medium [21] containing 1% of ethanol and 1% of acetic acid. The sampling was going on from March to May 2017. The cultures were incubated at 30 °C for 3 days at high humidity. The colonies were streaked several times to obtain pure cultures. Morphologically different colonies were preserved in medium with 20% glycerol at −80 °C for further analysis. 

### 2.2. 16. S-23S rRNA Gene ITS and 16S rRNA Gene Sequencing

With the aim to tentatively identify the preserved strains, the isolates were revitalized from −80 °C on RAE medium with 1% of ethanol and 1% of acetic acid. A single colony from each culture was inoculated onto the same medium and grown for three days to obtain enough biomass for DNA isolation. After biomass harvesting, the DNA was isolated by the GeneJET Genomic DNA Purification Kit (Thermo Scientific, Waltham, MA, USA). The 16S–23S rRNA gene ITS regions were amplified by the primers SpaFw (5′-TGCGG(T/C)TGGATCACCTCCT-3′) and SpaRev (5′-GTGCC(A/T)AGGCATCCACCG- 3′), and the 16S rRNA gene sequence by the primers 27f (5′-AGAGTTTGATCMTGGCTCAG-3′) and rH1542 (5′-AAGGAGGTGATCCAGCCGCA-3′). The PCR-products were purified with the GenJet PCR Purification Kit (Thermo Scientific, Waltham, Massachusetts, USA) and sequenced by the Sanger method at Microsynth (Vienna, Austria).

### 2.3. Genome Sequencing

Genomic DNA was extracted with the NucleoSpin Tissue kit (Macherey-Nagel, Düren, Germany) using the manufacturer’s protocol. Whole genome sequencing analysis was performed at the Medical Faculty of the University of Maribor using the Illumina MiSeq Platform. At the beginning, the paired-end libraries (2 × 250 bp) were prepared with the Nextera XT DNA Library Preparation Kit (Illumina, San Diego, CA, USA) following the manufacturer’s protocol. The sequencing was performed using MiSeq Reagent Kit v. 3 (600 cycles) (Illumina, San Diego, California, USA). The raw sequencing reads were quality checked and trimmed with Trimmomatic [22] following the genome assembly of the raw reads with Velvet [23].

### 2.4. Phenotypic Analysis

Phenotypic characteristics of the novel strains AV382 and AV436 were compared to those of type strains of closely related species of the *Komagataeibacter* genus. The strains were routinely precultured on RAE medium supplemented with 1% ethanol and 1% acetic acid, with the exception of *K. xylinus* LMG 1515^T^ which was grown on GY agar medium (5% glucose, 0.5% yeast extract and 1.5% agar). The plates were incubated aerobically at 30 °C for 3 days. The type strain of *Gluconacetobacter entanii* could not be included in the analysis since it is not available from any publicly available culture collection. All tests were performed as described previously by Škraban et al. [13] with the exception of gluconic acids identification for which a modified method of Gosselé et al. [24] was used. Briefly, the strains were grown on GY agar for 5 days, then a colony was precultured into liquid medium with 2% glucose and 2% Na-gluconate and grown with shaking (180 rpm) at 30 °C for 11 days. For separation of keto-gluconic acids, silica gel 60 thin layer chromatographic plates (Merck Millipore, Burlington, Massachusetts, USA) and a mobile phase composed of ethyl acetate, acetic acid, methanol and water in ratio 6:1.5:1.5:1 were used. After separation, the keto-acids were visualized with 2% suspension of diphenylamine.

### 2.5. Fatty Acid Analysis

The whole cell fatty acids of strains AV382 and AV436 were determined from cultures grown on two different media: GY agar medium and RAE medium supplemented with 1% ethanol and 1% of acetic acid. The strains were grown for 48 h at 30 °C under aerobic conditions. Inoculation and harvesting of the cells, extraction and analysis of the fatty acid methyl esters were performed according to the recommendations of the commercial identification system MIDI (Sherlock Microbial Identification System, Inc., Newark, DE, USA). The fatty acid methyl esters were separated by gas chromatography and identified using the aerobe database RTSBA6 (Sherlock v. 6.1)

### 2.6. Antimicrobial Resistance

For testing antimicrobial resistance of strains AV382 and AV436, a disc diffusion method was applied following a modified Kirby-Bauer method [25]. Briefly, the strains were grown on RAE medium with 1% ethanol and 1% acetic at 30 °C and high humidity for 3 days. Then the biomass was harvested, suspended in 0.9% NaCl and adjusted to a turbidity of McFarland standard 0.5 [26]. A swab was dipped into the inoculum tube and streaked over the surface of a RAE plate containing 1% ethanol and 1% acetic acid to enable confluent bacterial growth. Commercial discs impregnated with ampicillin (10 µg), chloramphenicol (30 µg), gentamicin (10 µg) and gentamicin (30 µg) were placed on the surface of agar. The plate was incubated for 2 days at 30 °C and high humidity before reading the inhibition zones.

### 2.7. Bioinformatics

*Komagataeibacter* phylogeny was constructed using concatenated core genes, 16S-23S rRNA gene ITS regions and 16S rRNA gene sequences.

The procedure started with local annotation of genome sequences by Prokka [27], followed by alignment at the minimum BlastP [28] identity of 90% by Roary [29]. From the concatenated core genes the phylogeny was inferred with the maximum likelihood algorithm included in PhyML [30] using the GTR nucleotide substitution model and 1000 bootstrap replicates.

When using 16S rRNA gene sequences and 16S-23S rRNA gene ITS, the homologous sequences of *Komagataeibacter* type strains and strains AV386 and AV436 were aligned by ClustalX [31]. The aligned sequences were used for phylogenetic tree construction by MEGA X [32]. All positions containing gaps were eliminated. Maximum-likelihood phylogenies were constructed based on the Tamura–Nei model [33] and 1000 bootstrap replicates.

Genes and operons important for the *Komagataeibacter* ecology, such as alcohol and aldehyde dehydrogenase genes (*adhA*, *aldh*), acetic acid resistance conferring genes (*aarC*, *azr1*, *aatA)* and extracellular polysaccharide synthesis operons (*bcs*, *ace*, *rfb*), were searched for by using BlastP [28]. The queries were protein products of these genes which were already identified in other *Komagateibacter* species spanning the diversity in this genus [13]. The results were checked manually for high similarity, coverage, operon structure as well as protein family membership included in Pfam [34] and CAZy [35].

Average nucleotide identity (ANI) values were calculated by the programme JSpecies [36]. For each sequenced genome, contigs longer than 500 bp were extracted and used for ANI calculation.

Genome distances between strains AV382, AV436 and type strains of species of *Komagataeibacter* were calculated by the Genome-to-Genome Distance Calculator 2.1. The method reliably mimics conventional DNA-DNA hybridization [37].

## 3. Results and Discussion

### 3.1. Isolation and Basic Characterization of Strains AV382 and AV436

Since vinegar is the most well-known product of acetic acid bacteria, it is not surprising that out of 17 type species of the genus *Komagataeibacter*, seven species, namely *Komagataeibacter europaeus* [38], *K. hansenii* [39], *Komagataeibacter kakiaceti* [40], *Komagataeibacter maltaceti* [12], *Komagataeibacter medellinensis* [8], *Komagataeibacter oboediens* [41] and *K. pomaceti* [13] have been isolated from vinegars. The novel strains AV382 and AV436 were also isolated from vinegar on RAE medium supplemented with 1% of acetic acid and 1% of ethanol after sampling the same bioreactor, but different batches for apple cider vinegar production. During apple cider vinegar production apple cider prepared from different varieties of apples is added to the bioreactor as a substrate. The apples are carriers of different acetic acid bacterial strains, which proceed to the vinegar producing bioreactors after resisting stress conditions during ethanol fermentation. This may be a reason of isolating the strains AV382 and AV436 from different batches but the same vinegar producing bioreactor. Although isolation and cultivation of acetic acid bacteria from industrial bioreactors for vinegar production have very low efficiency [20,21,38,42], both strains grew very well on RAE medium, but also on media without acetic acid and ethanol, such as GY and mannitol agar. The nutrient agar medium without any sugar supplement enabled only very poor and slow growth of both strains, which finally resulted in growth arrest. Both strains oxidize ethanol to acid and further on to CO_2_ and H_2_O on Carr and Frateur agar media. This is one of the typical characteristics of the genus *Komagataeibacter* [43]. Moreover, both strains exhibit high acetic acid tolerance by persisting up to 5% of acetic acid in liquid RAE medium with 1% and 3% of ethanol, which is comparable to the acidity during apple cider vinegar production. Both strains preserved the acetic acid tolerance after storage at −80 °C in the presence of 20% glycerol for at least one and half year.

The classical microbiological analysis showed that strains AV382 and AV436 are Gram-staining negative, catalase positive, oxidase negative and strictly aerobic bacteria. On RAE medium with 1% of acetic acid and 1% of ethanol both strains are forming round shaped colonies of irregular size between 0.9–3.0 mm in diameter after three days of cultivation. Microscopical examination showed small rods for both strains (Supplementary Figure S1). Cells of AV436 typically form short chains of 3–10 cells. The formation of chains was not observed for strain AV382.

### 3.2. Phylogenetic Affiliation of Strains AV382 and AV436

**Figure 1:**
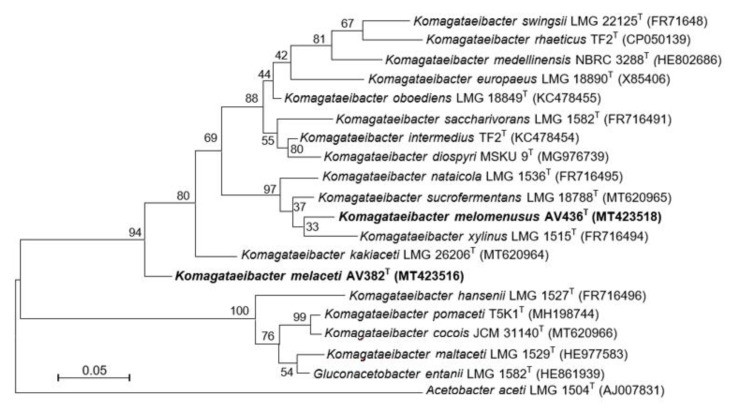
Phylogenetic reconstruction based on 16S–23S rRNA gene internal transcribed spacer (ITS) sequences of the type strains of species of the *Komagataeibacter* genus. Strains AV382 and AV436 representing novel spcies are in bold. The tree was constructed using the maximum-likelihood method. Bootstrap values are indicated at branching (1000 replicates). GenBank accession numbers are given in brackets and the scale bar represents the number of substitutions per nucleotide position.

The 16S rRNA gene sequencing confirmed the affiliation of AV382 and AV436 to the genus *Komagataeibacter* (Supplementary Figure S2). Besides, the nucleotide identity of both strains showed very high 16S rRNA gene sequence similarity to the closest species, representing just a few nucleotide differences. The 16S rRNA gene sequence of strain AV436 showed the highest similariry (99.4–99.8%) to the sequences of species *Komagataeibacter intermedius*, *Komagataeibacter oboediens*, *Komagataeibacter swingsii*, *K. nataicola*, *Komagataeibacter sucrofermentans*, *Komagataeibacter europaeus*, *Komagataeibacter diospyri* and *K. xylinus* and the sequence of strain AV382 the highest similarity (99.3–99.5%) to species *K. swingsii*, *K. europaeus*, *K. sucrofermentans, K. nataicola*, *Komagataeibacter kakiaceti*, *K. saccharivorans*, *K. oboediens* and *K. intermedius*. Since this high sequence identity represents only 2–9 nucleotide differences, we proceeded with the analysis of 16S–23S rRNA gene ITS regions which revealed more nucleotide differences to type strains of species of the genus *Komagataeibacter*. The closest relatives based on comparison of 16S–23S rRNA gene ITS was *Komagataeibacter kakiaceti* (91.6%) for strain AV382, and species *K. xylinus* (93.9%), *K. sucrofermentans* (92.7%) and *K. nataicola* (94.6%) for strain AV436. The phylogenetic relation among all type strains of the species of *Komagataeibacter* and strains AV382 and AV436, based on 16S–23S rRNA gene ITS is shown in Figure 1.

**Figure 2:**
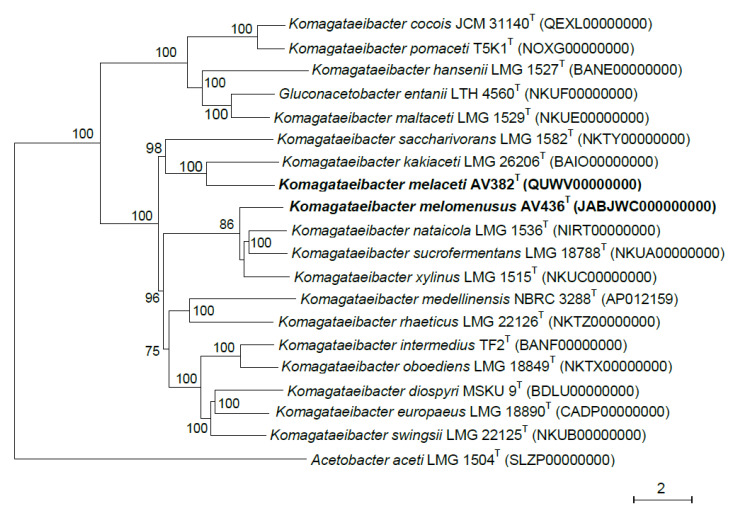
Phylogenetic reconstruction based on core genes (46 at minimum BlastP identity of 90%) of the type strains of species of the *Komagataeibacter* genus. Strains AV382 and AV436 representing novel species are in bold. The tree was constructed using the maximum-likelihood method. Bootstrap values are indicated at branching (1000 replicates). GenBank accession numbers are given in brackets and the scale bar represents 2% estimated sequence differences.

The genome sequencing of strains AV382 and AV436 enabled us to perform further phylogenetic analysis. We constructed a phylogenetic tree based on core genes (Figure 2) which confirmed the classification of strain AV382 close to *K. kakiaceti* and strain AV436 close to *K. xylinus*, *K. sucrofermentans* and *K. nataicola*. Moreover, the ANI values calculated from the comparison of the entire genome sequences of both strains to the type strains species of the *Komagataeibacter* genus (Supplementary Table S1) revealed that strains AV382 and AV436 represent novel species of this genus since the highest ANI value (91.6%) was below the 94% cut-off value for species delineation [44]. The novel species status of both strains AV382 and AV436 was confirmed by *in-silico* DNA-DNA hybridization calculations (Supplementary Table S2) since both strains showed ≤47.3% identity to type strains of species of the *Komagataeibacter* genus which is below the accepted value of 70% for species delineation [45].

### 3.3. Phenotypic Characterization of Strains AV382 and AV436

The fatty acid profile of both strains, AV382 and AV436 was analyzed from cells cultured on RAE and GY medium (Table 1). In both cases the major fatty acid was *cis*-vaccenic acid (C_18:1_ *ω*7c) with 34.4–36.9 mol% for strain AV382 and 31.4–43.4 mol% for strain AV436. This fatty acid was reported as the predominant fatty acid for all acetic acid bacteria, with the exception of the recently described species *Komagataeibacter cocois* JCM 31140^T^ [9]. However, the fatty acid analysis of this type strain cultured on GY and RAE medium could not confirm the published results (Table 1). Interestingly, in the strain AV382, a second predominant fatty acid was a 19-carbon cyclopropane analogue of *cis*-vaccenic acid, i.e., C_19:0_ cyclo *ω*8c. This fatty acid represents 13 mol% of the total fatty acids when the strain was grown on RAE medium and 16.7 mol% when grown on GY medium. Cyclopropane fatty acids as the major fatty acids are not typical for the genus *Komagataeibacter* and also not for other genera of acetic acid bacteria [46], with the exception of the genera *Swaminathania* [47], *Saccharibacter* [48] and *Bombella* [49]. The presence of cyclopropane fatty acid in bacteria favours tolerance to various stressors, such as osmotic and low pH [50]. The fatty acid C_19:0_ cyclo *ω*8c is synthesized from monounsaturated fatty acids; for example, from *cis*-vaccenic acid [51]. The high amount of this cyclopropane fatty acid in the novel strain AV382 can be used as a differentiation marker from other species of the genus *Komagataeibacter*.

Both strains AV382 and AV436 showed resistance against chloramphenicol (30 µg), the strain AV436 additionally to gentamicin (10 µg). However, both strains showed inhibition zones around ampicillin (10 µg) and gentamicin (30 µg). Using the Comprehensive Antibiotic Resistance Database [52] for analysis of possible antibiotic resistance molecular determinants in genomes of both strains, chloramphenicol acetyltransferase homologue were identified: RFD20163 for strain AV382 and WP_172158671 for strain AV436. These enzymes are structurally modifying the antibiotic and thus inhibiting chloramphenicol catalytic activity [53], which may explain resistance against chloramphenicol of both strains. No homologue to proteins coding for aminoglycoside acetyltransferases which are responsible for aminoglycoside antibiotic inactivation, such as gentamicin [54], could be identified in both genomes. However, in both genomes some putative efflux resistance-modulation-cell (RND) permease transporters, DHA2 family efflux major facilitator superfamily (MFS) permease transporters and two multidrug efflux small multidrug resistance (SMR) transporters have been identified (Supplementary Table S3). These proteins may be involved in transport of antibiotics out of the cell [55]. The presence of antibiotic resistance determinants in food grade bacteria, such as acetic acid bacteria, is not problematic by itself, but when this genetic information is transferred to human pathogenic bacteria, it may become of immense concern. The probability of this scenario is presently increasing with a trend of preparing and consuming nonfiltered biomass containing apple cider vinegar.

Further phenotypic analysis showed that both strains produce 2-keto-D-gluconic and 5-keto-D-gluconic acids from glucose which differentiates strain AV382 from its closest relative *K. kakiaceti* and strain AV436 from one of the three most closest relatives *K. sucrofermentans* (Table 2). None of the strains produced 2,5-diketo-D-gluconic acid. Both strains formed 2-keto-D-gluconic acid and 5-keto-D-gluconic acid during cultivation in glucose rich liquid medium. In contrast to strain AV382, 2-keto-D-gluconic acid could not be detected any more in the growth medium of strain AV436 at our second sampling point of cultivation, suggesting that the acid was transported back into the cytoplasm and used as an energy source. This kind of metabolic action was observed in some other acetic acid bacteria [56]. None of the strains AV382 and AV436 grew in the presence of 30% of glucose. This characteristic obviously differentiates strain AV382 from both closest species *K. kakiaceti* and *K. saccharivorans* (Table 2). Strain AV436 can well use D-ribose, sorbitol, D-mannitol and glycerol as carbon source and also 1-propanol, albeit with weak growth. In contrast, *K. nataicola* cannot grow on 1-propanol. Strain AV382 can grow on Hoyer-Frateur medium with ethanol as carbon source, but both of its closest relatives *K. kakiaceti* and *K. saccharivorans* cannot. In contrast to AV382 and *K. xylinus*, strain AV436 cannot grow on Hoyer-Frateur medium with ethanol as carbon source. Strain AV382 can also grow on Asai medium with ethanol, but its closest relative *K. kakiaceti* cannot. The pellet of strain AV436 did not dissolve after cooking in 5M NaOH, which shows that the strain is a cellulose producer. Cellulose production was not observed for the strain AV382. The tolerance to 5% acetic acid differentiates strain AV382 from *K. kakiaceti* and *K. saccharivorans* which tolerate 4% of acetic acid. The 5% acetic acid tolerance of AV436 differentiates it from *K. xylinus* and *K. nataicola*, which tolerate 4% of acetic acid in the presence of 1% ethanol and 3% of acetic acid in the presence of 3% ethanol.

### 3.4. Genome analysis of strains AV382 and AV436

Acetic acid bacteria in vinegar are under high acid stress, and thus they evolved tools for coping with acid. Among them is a transporter pumping acetic acid out of the cell, extracellular polysaccharide formation to block entrance of acid in the cytoplasm and major alcohol and aldehyde dehydrogenases embedded into the cytoplasmic membrane to oxidize ethanol to acetic acid and parallelly producing a big energy pool [57]. Using the available information on molecular determinants relevant to these characteristics [13], we searched the genome sequences of strains AV382 and AV436 for homologues.

**Figure 3:**
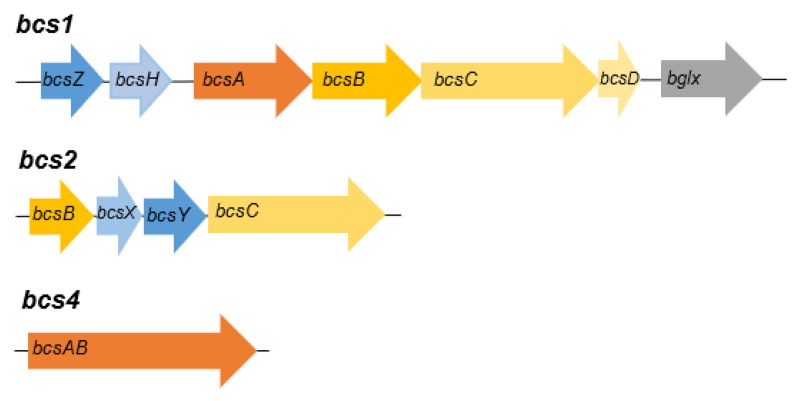
Structure of operons *bcs1*, *bcs2* and *bcs4* of *Komagataeibacter melomenusus* AV436^T^. The gene sizes are presented relatively to each other. Gene symbols are taken from publication of Römling and Galperin [62]. The gene symbols correspond to the following product: *bcsZ*, endoglucanase; *bcsH*, cellulose-complementing protein; *bcsA*, cellulose synthase catalytic subunit A; *bcsB*, periplasmic cellulose synthase subunit Bc; *bcsC*, cellulose synthase subunit C, spans periplasm and outer membrane; *bcsD*, periplasmic cellulose synthase subunit D; *bglx*, β-glucosidase; *bcsX,* acyltransferase; *bcsY*, hydrolase.

**Figure 4:**
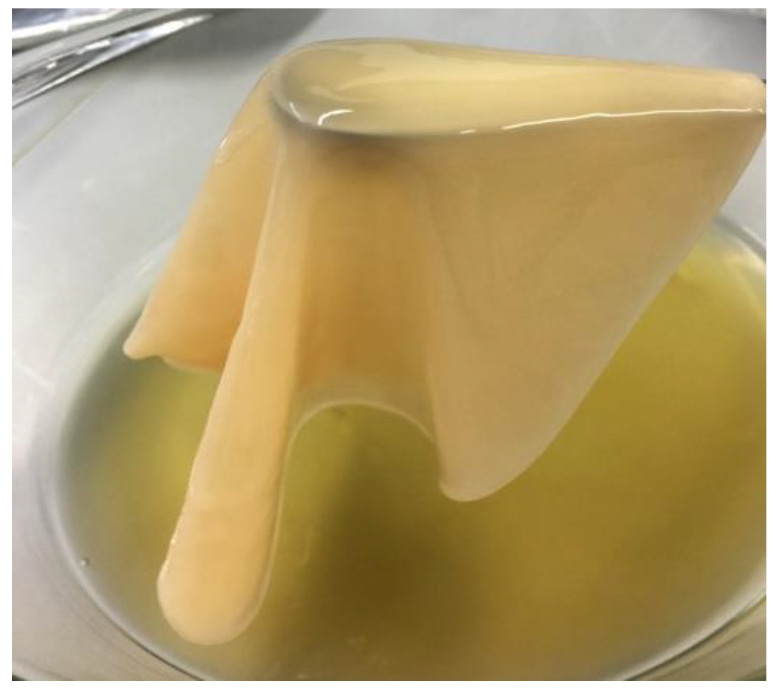
Production of cellulose with strain AV436 on RAE medium.

The genome sequence of AV436 is coding for homologues to proteins encoded by cellulose operon *bcs1* [58], *bcs2* [59], *bcs4* [60], but not to *bcs3* [61]. Genes of operon *bcs1* (Figure 3) are coding for putative endo-1,4-beta-glucanase (NPC65873), cellulose-complementing protein A (NPC65872), cellulose synthase catalytic subunit A (NPC65871), cellulose synthase subunit B (NPC65870), cellulose synthase subunit C (NPC65869), cellulose synthase subunit D (NPC65868) and β-glucosidase (NPC65867). Except for NPC65872, all other ORFs of putative AV436 *bcs1* are of similar size and almost identical to proteins encoded by *bcs1* of *Komagataeibacter xylinus* E25 [58]. The cellulose-complementing protein A acts on expression levels of BcsB and BcsC, interacts with BcsD and probably assists in arrangement of glucan chains into crystalline ribbons [62]. Since the NPC65872 and its homologue AHI24409 in *K. xylinus* E25 share only 36.7% identity, this may affect the quantity and the quality of cellulose production in each species. The AV436 *bcs2* operon (Figure 3) is encoding putative cellulose synthase subunit C (NPC65045), hydrolase (NPC65044) and acyltransferase (NPC65046). Besides the two operons, a catalytic subunit cellulose synthase (NPC67849) encompassing operon *bcs4* (Figure 3) has been identified at another chromosomal location of AV436. All these ORFs support our phenotypic observation on strong cellulose production by strain AV436. The strain produces cellulose layer of approximate size 25 cm × 15 cm × 0.3 cm on 500 mL RAE medium in 5 days (Figure 4). In the genome sequence of AV382, homologues to some of the proteins encoded by operon *bcs1* and *bcs2* were identified but again no homologues to proteins of operon *bcs3*. Genes coding for acetan, the second most well-known polysaccharide exported by some acetic acid bacteria [63,64], were then searched in AV382 and AV436. Homologues to some of the proteins encoded by acetan operon have been identified in strain AV382 (glycosyltransferase: WP_11670167, flipase: WP_116701683), but not in strain AV436. Both strains also possess homologues to proteins encoded by *rfbABCD* operon, which is involved in dTDP-rhamnose synthesis pathway and most probably forms capsular polysaccharides attached to the cell [65,66]. In strain AV436 the putative *rfbA* coding for glucose-1-phosphate thymidylyltransferase (WP_172156247) and *rfbB* coding for dTDP-glucose 4,6-dehydratase (WP_172156248) are part of an operon, the *rfbC* coding for dTDP-4-dehydrorhamnose 3,5-epimerase (WP_172156300) and *rfbD* (WP_172156302) coding for dTDP-4-dehydrorhamnose reductase are part of another operon. In strain AV382 the genes coding for the putative proteins RfbA (WP_116702174) and RfbB (WP_116702134) are not part of a transcriptional unit. In contrast to *rfbA and rfbB*, the genes coding for putative RfbC (WP_116702180) and RfbD (WP_116702181) are part of an operon.

AV382 and AV436 both possess AarC, RFD20455 and NPC66889, respectively, which is a homologue to succinyl-coenzyme A (CoA): acetate CoA transferase. This enzyme replaces succinyl-CoA synthetase and converts succinyl-CoA and acetate to acetyl-CoA and succinate, and thus enables an efficient assimilation of acetic acid [67]. In strain AV436, one MFS transporter (WP_172156362) shows some similarities to plasma membrane protein of *Saccharomyces cerevisiae* (Azr1), which is contributing cell adaptation to weak acids [68]. Further studies should reveal its potential involvement in acetic acid tolerance in *Komagataeibacter melomenusus*.

Of three putative types of a catalytic subunit of PQQ-dependent alcohol dehydrogenase identified in some *Komagataeibacter* species [13], only homologues to AdhA-1 have been identified in AV382 (RFD19113) and AV436 (NPC67328). In both strains, the homologues to AldH, a catalytic subunit of aldehyde dehydrogenase, have also been identified: WP_116702452 for AV382 and WP_172154413 for AV436.

In acetic acid bacteria, 2-ketogluconate and 5-ketogluconate are mainly produced from D-gluconate with membrane bound FAD-dependent gluconate dehydrogenase (FAD-GADH) and PQQ-dependent glycerol dehydrogenase (PQQ-GLDH), respectively [69]. Some strains have also PQQ-dependent gluconate dehydrogenase (PQQ-GADH) which converts D-gluconate to 2,5-diketogluconate [70]. In genomes of both strains, AV382 and AV436 the putative large and small subunits of FAD-GADH (AV382: WP_116702246, WP_116702271; AV436: WP_172157429, WP_172157431) and PQQ-GLDH (AV382: WP_116701675, WP_116701676; AV436: WP_172158295, WP_172158288) but not PQQ-GADH were identified which support the phenotypic data described above.

## 4. Conclusions

In this study we described two novel species from the group of acetic acid bacteria. Their main features are summarized below. The study was focused on taxonomic characterization of both novel strains, but it also confirms that the microbiota of apple cider vinegar is heterogenous as described in previous studies using different molecular approaches [71,72,73,74]. Additionally, the genetic potential of both strains confirms that the acetic acid bacteria are biotechnologically important microorganisms, which will remain in focus of further scientific studies.

### 4.1. Description of Komagataeibacter melaceti sp. nov.

*Komagataeibacter melaceti* (mel.a.ce’ti. G. n. melon, apple; L. n. acetum, vinegar, N.L. n. melaceti, of apple vinegar from where the type strain was isolated).

Cells are Gram-staining negative, rod shaped, 1.9–5.0 µm long, 1.1–1.5 µm wide, occurring singly. Colonies are cream to light brown, circular, smooth, forming convex circled shapes with diameters of about 1.0–3.0 mm on RAE medium supplemented with 1% ethanol and 1% acetic acid after 3 days of incubation at high humidity. Cells are obligately aerobic, catalase positive and oxidase negative. The optimum growth temperature is 28–30 °C. Ethanol is oxidized to acetic acid and further on to CO_2_ and H_2_O. Both 2- and 5-keto-D-gluconic acids are formed but not 2,5-diketo-D-gluconic acid from D-glucose. Bacterial cellulose is not produced from D- glucose. Growth is observed in the presence of 1–5% (*v/v*) acetic acid and 1% or 3% ethanol (*v/v*), but acetic acid is not required for growth. Growth is observed on glucose-yeast agar and mannitol agar. Growth is observed on D-ribose, D-glucose, D-mannitol, D-sorbitol, glycerol and 1-propanol as sole carbon source. The strain can utilize ammoniacal nitrogen in Hoyer-Frateur medium with D-glucose, D-mannitol and ethanol, and in Asai medium with D-glucose and D-mannitol, but not with ethanol. The major cellular fatty acid is C_18:1_ *ω*7*c* and a second predominant fatty acid is C_19:0_ cyclo *ω*8c.

The type strain, AV382^T^ (= ZIM B1054^T^ = LMG 31303^T^ = CCM 8958^T^), was isolated from apple cider vinegar in Kopivnik, Slovenia.

The GenBank/ENA/DDBJ accession number for the 16S rRNA gene sequence, 16S–23S rRNA gene ITS sequence and complete genome sequence of the type strain are MT422125, MT423516 and QUWV00000000, respectively.

### 4.2. Description of Komagataeibacter melomenusus sp. nov.

*Komagataeibacter melomenusus* (mel.o.me.nu’sus. G. n. melon, apple; Gr. v. meno to reside, stay, live [masc. pres. part. menusus]; N.L. masc. adj. melomenusus from apple [cider] as the niche of these bacteria).

Cells are Gram-staining negative, rod shaped, 1.5–2.8 µm long, 0.8–1.0 µm wide, occurring in chains of 3–10 cells. Colonies are cream to light brown, circular, smooth, forming convex circle shapes with diameter of about 0.9–3.0 mm on RAE medium supplemented with 1% ethanol and 1% acetic acid after 3 days of incubation at high humidity. Cells are obligately aerobic, catalase positive and oxidase negative. The optimum growth temperature is 28–30 °C. Ethanol is oxidized to acetic acid and further on to CO_2_ and H_2_O. Both 2- and 5-keto-D-gluconic acids are formed but not 2,5-diketo-D-gluconic acid from D-glucose. Bacterial cellulose is produced from D- glucose. Growth is observed in the presence of 1–5% (*v/v*) acetic acid and 1% or 3% ethanol (*v/v*), but acetic acid is not required for growth. Growth is observed on glucose-yeast agar and mannitol agar. Growth is observed on D-ribose, D-glucose, D-mannitol, D-sorbitol and glycerol as sole carbon source. Growth on 1-propanol is weak. The strain can utilize ammoniacal nitrogen in Hoyer-Frateur medium with D-glucose and D-mannitol, but not with ethanol, in Asai medium with D-glucose and D-mannitol, but not with ethanol. The major cellular fatty acid is C_18:1_ *ω*7*c*.

The type strain, AV436^T^ (= ZIM B1056^T^ = LMG 31304^T^ = CCM 8959^T^), was isolated from apple cider vinegar in Kopivnik, Slovenia.

The GenBank/ENA/DDBJ accession number for the 16S rRNA gene sequence, 16S–23S rRNA gene ITS sequence and complete genome sequence of the type strain are MT422127, MT423518 and JABJWC000000000, respectively.

## Figures and Tables

**Table 1:** Cellular fatty acid profiles of strains AV382, AV436 and *Komagataeibacter cocois* JCM 31140^T^.

Fatty Acid	AV382 (mol%)	AV436 (mol%)	*K. cocois* JCM 31140^T^ (mol%)
C_14:0_ 2-OH	5.9/6.5	13.2/1.7	9.5/5.5
C_16:0_	9.2/9.0	16.4/2.0	13.8/1.7
C_16:0_ 2-OH	7.1/7.4	15.2/2.6	8.0/6.3
C_16:0_ 3-OH	1.1/1.7	5.4/2.0	1.7/1.7
C_17:0_	8.2/6.7	0.8/1.0	nd/1.9
C_17:1_ *ω*6*c*	6.6/2.6	nd/0.4	nd/0.9
C_18:1_ *ω*7*c*	36.9/34.4	31.4/43.4	58.9/61.9
C_19:1_ cyclo *ω*8*c*	13.0/16.7	5.0/4.5	nd/nd

For each fatty acid two numbers are presented: first one for biomass harvested from RAE agar with 1% acetic acid and 1% ethanol, the second one for biomass harvested from GY agar. The fatty acids with more than 3 mol% in at least one of the strains are presented. nd, not detectable.

**Table 2:** Differential phenotypic characteristics of strains AV382 and AV436 from their closest species.

Phenotypic Features	1	2	3	4	5	6	7
Formation from D-Glucose							
2-keto-D-Gluconic acid	+	+	+ ^a^	+ ^a^	+ ^b^	+ ^a^	- ^c^
5-keto-D-Gluconic acid	+	+	+ ^a^	- ^a^	+ ^b^	+ ^a^	- ^c^
Growth on carbon sources:							
D-Ribose	+	+	+	+	w	+	w
Sorbitol	+	+	+	+	w	+	w
D-Mannitol	+	+	+	+	+	+	+
Glycerol	+	+	+	+	+	+	w
1-Propanol	+	w	+	+	-	w	+
Growth in the presence of 30% D-glucose	-	-	-	-	-	+	+
Utilization of ammoniacal nitrogen in:							
Hoyer-Frateur medium with:							
Ethanol	+	-	+	-	-	-	-
Asai medium with:							
D-Mannitol	+	+	+	+	+	+	-
Growth on RAE medium in the presence of 1% ethanol and acetic acid at:							
4%	+	+	+	+	+	+	+
5%	+	+	-	+	-	-	+
Growth on RAE medium in the presence of 3% ethanol and acetic acid at:							
4%	+	+	-	+	-	+	+
5%	+	+	-	+	-	-	-

Strains: 1, AV382^T^; 2, AV436^T^; 3, Komagataeibacter xylinus LMG 1515^T^; 4, Komagataeibacter sucrofermentans LMG 18788^T^; 5, Komagataeibacter nataicola LMG 1536^T^; 6, Komagataeibacter saccharivorans LMG 1582^T^; 7, Komagataeibacter kakiaceti LMG 26206^T^. Data marked with letters were obtained from publications: a, Castro et al. [8]; b, Slapšak et al. [12] and c, Iino et al. [40]. Abbreviations: +, positive growth; - negative growth; w, weak growth.

## Supplementary Materials

The following are available online at https://www.mdpi.com/2076-2607/8/8/1178/s1, Figure S1: Microscopic images of bacterial cells after Gram-staining at 1000-fold magnification. A: *Komagataeibacter melaceti* AV382^T^, B: *Komagataeibacter melomenusus* AV436^T^, Figure S2: Phylogenetic reconstruction based on 16S rRNA gene sequences of the type strains of the species of *Komagataeibacter* genus. The tree was constructed using the maximum-likelihood method. Bootstrap values are indicated at branching (1000 replicates). *Acetobacter aceti* is included as outgroup. GenBank accession numbers are given in brackets and the scale bar represents the number of substitutions per nucleotide position, Table S1: ANIb (A) and ANIm (B) values for strains AV382 and AV436 in comparison to the type strains of species of the *Komagataeibacter* genus, Table S2: Digital DNA-DNA hybridization values of strains AV382 and AV436 in comparison to type strains of species of genus *Komagataeibacter*, Table S3: List of putative efflux protein subunits in genomes of strains AV382 and AV436.
