# Supplementary material for: Description of Komagataeibacter melaceti sp. nov. and Komagataeibacter melomenusus sp. nov. Isolated from Apple Cider Vinegar
Source: Microorganisms. 2020 Aug 3;8(8):1178. doi: 10.3390/microorganisms8081178 (PMC7465234; doi:10.3390/microorganisms8081178)
Supplement: Supplementary file 1 [file microorganisms-08-01178-s001.pdf]

Supplementary Materials

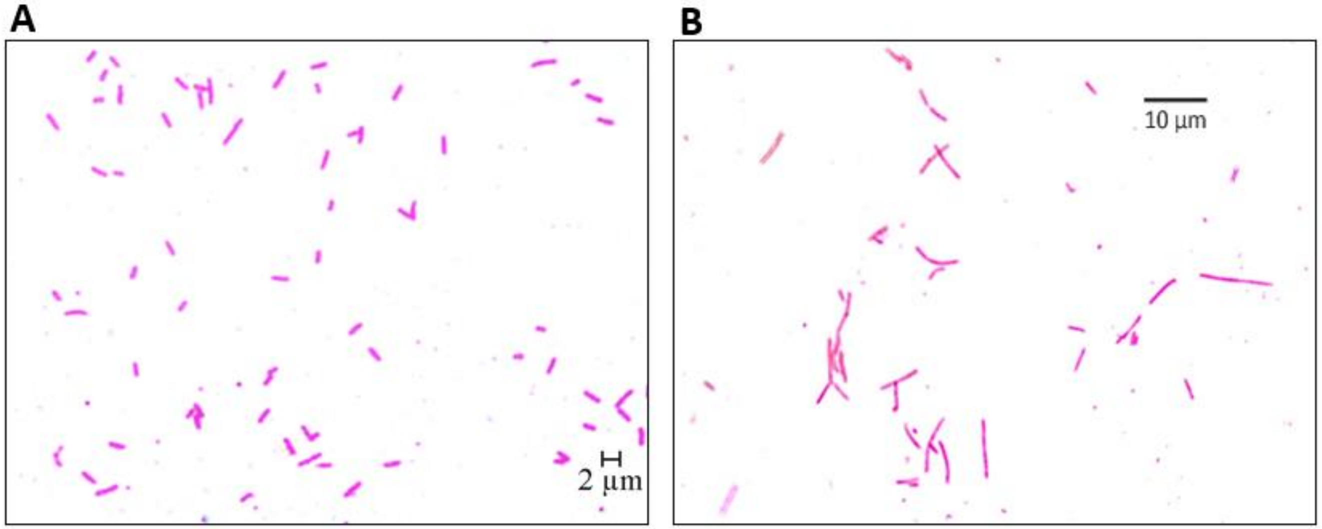

Supplementary Figure S1: Microscopic images of bacterial cells after Gram-staining at 1000-fold magnification. A: *Komagataeibacter melaceti* AV382^{T}, B: *Komagataeibacter melomenusus* AV436^{T}.

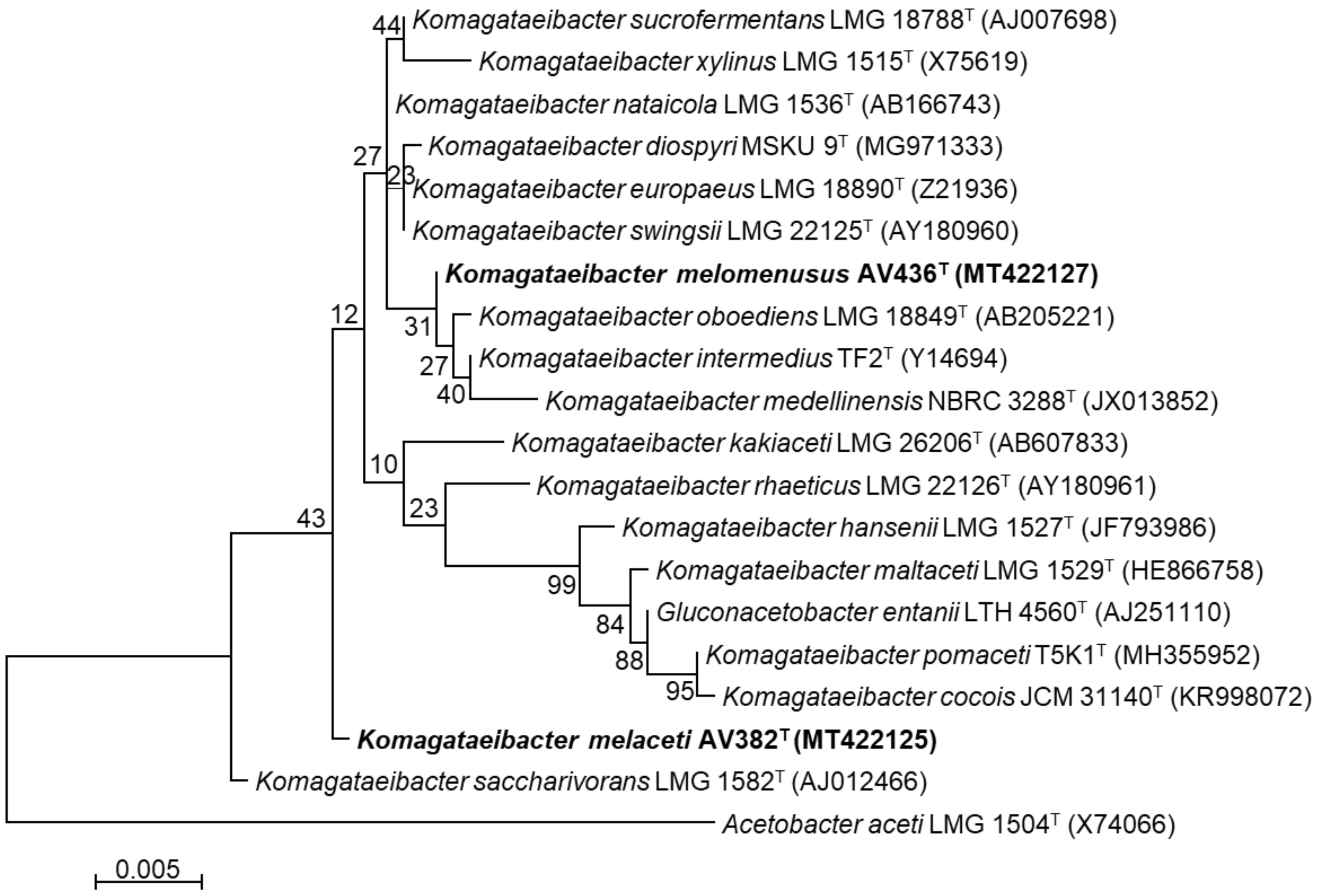

Supplementary Figure S2: Phylogenetic reconstruction based on 16S rRNA gene sequences of the type strains of species of *Komagataeibacter* genus. The tree was constructed using the maximum-likelihood method. Bootstrap values are indicated at branching (1000 replicates). *Acetobacter aceti* is included as outgroup. GenBank accession numbers are given in brackets and the scale bar represents the number of substitutions per nucleotide position.

Supplementary Table 1: ANIb (A) and ANIm (B) values for strains AV382 and AV436 in comparison to the type strains of species of the *Komagataeibacter* genus.

A

	1	2	3	4	5	6	7	8	9	10	11	12	13	14	15	16	17	18	19
1 *Komagataeibacter* sp. AV382	*	82.6	81.4	75.4	77.5	83.1	75.6	82.9	88.2	77.9	80.7	82.3	82.7	81.9	82.8	82.6	83.3	77.9	82.4
2 *Komagataeibacter* sp. AV436	82.5	*	81.7	75.4	78.3	83.2	75.8	82.9	83.3	78.3	81.3	90.9	82.9	82.9	81.0	91.5	83.5	77.8	91.5
3 *Komagataeibacter diospyri*	83.4	83.5	*	77.0	76.7	90.9	75.9	89.3	83.8	77.1	82.9	82.8	88.5	83.7	83.5	82.4	91.8	76.7	82.4
4 *Komagataeibacter cocois*	77.0	76.9	77.1	*	85.6	77.1	84.2	77.1	77.5	85.6	75.9	76.5	76.8	76.7	76.7	76.5	77.3	95.6	76.4
5 *Gluconacetobacter entanii*	77.5	78.1	75.7	83.1	*	78.7	85.8	78.0	78.8	91.2	75.8	77.5	78.1	77.2	76.6	77.4	78.4	84.3	77.5
6 *Komagataeibacter europaeus*	83.5	83.7	87.2	75.4	79.3	*	76.7	88.7	83.7	79.2	81.6	83.4	89.7	83.5	82.3	83.4	91.3	80.2	84.5
7 *Komagataeibacter hansenii*	75.5	76.1	75.5	82.6	85.7	76.4	*	76.3	76.5	85.4	75.3	75.9	75.7	75.8	75.5	75.6	75.9	82.1	75.7
8 *Komagataeibacter intermedius*	84.6	84.2	85.9	74.8	80.5	89.5	76.2	*	84.5	79.9	81.5	84.4	93.9	82.9	83.4	84.0	89.5	81.2	84.9
9 *Komagataeibacter kakiaceti*	88.4	83.2	81.9	75.9	78.6	83.5	76.1	83.1	*	78.3	81.0	82.4	82.9	82.4	83.3	82.5	83.2	78.2	82.4
10 *Komagataeibacter maltaceti*	78.1	78.4	76.4	83.8	91.3	78.4	85.4	78.5	78.5	*	76.9	78.1	77.9	77.1	76.6	78.5	78.9	84.6	78.1
11 *Komagataeibacter medellinensis*	80.9	81.7	81.0	74.9	76.1	82.0	74.9	82.0	81.4	76.9	*	81.6	81.8	86.6	80.4	81.6	82.2	75.3	81.3
12 *Komagataeibacter nataicola*	82.2	91.1	81.7	75.9	77.4	82.9	75.5	83.1	82.4	78.1	81.4	*	82.7	82.3	81.0	91.9	83.4	77.7	91.3
13 *Komagataeibacter oboediens*	82.7	82.6	85.7	75.3	78.5	89.3	76.1	93.8	82.7	77.9	81.4	82.7	*	82.9	82.1	82.4	88.3	78.9	83.5
14 *Komagataeibacter rhaeticus*	81.6	82.9	81.6	76.0	77.3	83.5	75.5	82.9	82.1	77.2	85.9	82.3	83.1	*	81.2	82.7	83.3	76.7	82.9
15 *Komagataeibacter saccharivorans*	82.7	81.2	81.3	75.1	76.5	82.3	75.5	82.7	83.3	76.4	80.2	81.1	82.0	81.4	*	81.0	82.1	76.4	81.0
16 *Komagataeibacter sucrofermentans*	82.6	91.7	80.8	75.5	77.4	83,0	75.3	83.1	82.8	78.2	81.5	92.3	82.6	82.7	81.1	*	83.5	77.6	91.7
17 *Komagataeibacter swingsii*	83.0	83.4	88.8	76.1	78.2	91.1	75.9	88.6	82.8	78.5	81.7	83.3	88.1	83.2	81.8	83.3	*	79.1	83.6
18 *Komagataeibacter pomaceti*	78.5	78.3	75.3	92.3	84.5	78.9	82.2	78.9	78.9	84.8	75.2	78.0	78.2	76.7	76.7	78.0	79.4	*	78.4
19 *Komagataeibacter xylinus*	82.3	91.4	80.5	75.3	77.6	83.4	75.4	82.9	82.3	77.6	81.2	90.9	83.2	82.8	81.1	91.3	83.6	78.1	*

B

	1	2	3	4	5	6	7	8	9	10	11	12	13	14	15	16	17	18	19
1 *Komagataeibacter* sp. AV382	*	86.6	86.0	85.1	86.1	86.7	84.5	86.8	90.0	86.6	85.1	86.2	86.2	85.9	85.9	86.5	86.8	87.0	86.3
2 *Komagataeibacter* sp. AV436	86.6	*	86.4	84.9	86.9	86.9	85.1	86.7	87.1	86.6	85.6	92.2	86.3	86.6	85.4	92.6	86.9	86.9	92.7
3 *Komagataeibacter diospyri*	86.0	86.4	*	84.9	84.9	92.0	84.8	90.4	86.4	85.2	86.3	86.1	89.9	86.6	86.2	86.2	92.6	84.8	86.2
4 *Komagataeibacter cocois*	85.1	84.9	84.9	*	87.6	85.3	86.9	85.0	85.2	87.7	84.8	85.0	84.9	85.5	85.3	85.0	85.2	95.7	85.0
5 *Gluconacetobacter entanii*	86.2	86.9	84.9	87.6	*	86.9	88.0	86.8	86.9	92.3	84.5	86.2	86.4	85.4	85.2	86.4	86.2	87.6	85.9
6 *Komagataeibacter europaeus*	86.7	86.9	92.0	85.3	86.9	*	85.5	90.3	87.3	86.7	86.0	86.6	90.6	86.7	86.1	86.6	92.2	87.4	87.0
7 *Komagataeibacter hansenii*	84.5	85.1	84.8	86.9	88.0	85.5	*	85.1	84.8	87.8	84.5	85.2	84.7	84.8	84.9	85.0	84.4	86.1	84.9
8 *Komagataeibacter intermedius*	86.8	86.7	90.4	85.0	86.8	90.3	85.1	*	87.1	87.2	85.8	86.9	94.7	86.4	86.5	86.9	90.4	88.2	86.9
9 *Komagataeibacter kakiaceti*	90.0	87.1	86.4	85.2	86.9	87.3	84.8	87.1	*	86.6	85.5	86.5	86.8	86.5	86.6	86.6	87.1	86.7	86.4
10 *Komagataeibacter maltaceti*	86.6	86.8	85.2	87.7	92.2	86.7	87.8	87.2	86.6	*	86.6	86.6	86.3	85.3	85.2	87.1	86.7	87.9	86.2
11 *Komagataeibacter medellinensis*	85.1	85.6	86.3	84.8	84.5	86.0	84.5	85.8	85.5	86.6	*	85.6	85.7	88.1	85.0	85.5	85.9	84.7	85.5
12 *Komagataeibacter nataicola*	86.2	92.2	86.1	85.0	86.2	86.6	85.2	86.9	86.5	86.6	85.6	*	86.2	86.2	85.3	93.0	86.9	86.9	92.3
13 *Komagataeibacter oboediens*	86.2	86.3	89.9	84.9	86.4	90.6	84.7	94.7	86.8	86.2	85.7	86.2	*	86.4	85.8	86.2	89.7	86.7	86.6
14 *Komagataeibacter rhaeticus*	85.9	86.6	86.6	85.5	85.4	86.7	84.8	86.4	86.5	85.2	88.1	86.2	86.4	*	85.5	86.3	86.7	85.1	86.5
15 *Komagataeibacter saccharivorans*	85.9	85.4	86.2	85.3	85.2	86.1	84.9	86.5	86.6	85.2	85.1	85.3	85.8	85.5	*	85.3	85.9	85.0	85.3
16 *Komagataeibacter sucrofermentans*	86.5	92.6	86.2	85.0	86.4	86.6	85.0	86.9	86.6	87.1	85.5	93.0	86.2	86.3	85.3	*	86.9	87.2	92.5
17 *Komagataeibacter swingsii*	86.8	86.9	92.6	85.2	86.2	92.2	84.5	90.3	87.1	86.7	85.9	86.9	89.7	86.7	85.9	86.9	*	87.6	87.0
18 *Komagataeibacter pomaceti*	87.0	86.9	84.8	95.7	87.6	87.4	86.1	88.2	86.7	87.9	84.7	86.9	86.7	85.1	85.1	87.2	87.6	*	87.4
19 *Komagataeibacter xylinus*	86.3	92.7	86.2	85.0	85.9	87.0	84.9	86.9	86.4	86.3	85.5	92.3	86.7	86.5	85.3	92.5	87.0	87.4	*

Supplementary Table 2: Digital DNA-DNA hybridization values of strains AV382 and AV436 in comparison to type strains of species of genus *Komagataeibacter*.

	1	2	3	4	5	6	7	8	9	10	11	12	13	14	15	16	17	18	19	20
1 *Komagataeibacter* AV382	-	27.3	22.8	28.2	21,0	28,0	37.7	21.1	23.7	24.5	26.5	27.1	26,0	26.7	26.9	28.2	23.8	26.7	21.5	27.3
2 *Komagataeibacter* AV436	27.3	-	23.4	28.6	21.4	28.1	29.5	21.1	23.5	25.4	45.4	27.2	27.4	25.2	46.9	28.3	24,0	47.3	22.2	21.8
3 *Gluconacetobacter entanii*	22.8	23.4	-	24.8	31.8	24.1	26,0	31.7	45.5	21.1	23,0	23.7	21.9	21.7	22.7	23.5	29.1	22.6	29.1	21.6
4 *Komagataeibacter europaeus*	28.2	28.6	24.8	-	22.1	39.3	30.2	21.3	24.5	26.3	28,0	40.6	28.2	26.6	28.1	46.1	26.1	29.3	22.3	43.7
5 *Komagataeibacter hansenii*	21,0	21.4	31.8	22.1	-	21.4	22.5	90.7	31.3	20.7	21.5	21.1	20.9	21.3	21.1	21,0	26.4	21.4	28.4	21.2
6 *Komagataeibacter intermedius*	28,0	28.1	24.1	39.3	21.4	-	29.6	21.2	24.2	26,0	28.1	57.7	27.6	27.2	28,0	38.9	25.3	28.3	21.5	37.8
7 *Komagataeibacter kakiaceti*	37.7	29.5	26,0	30.2	22.5	29.6	-	22.2	25.6	26.5	28.3	28.8	28,0	28.8	28.3	29.4	25.7	27.9	22.5	29.1
8 *Komagataeibacter kombuchae*	21.1	21.1	31.7	21.3	90.7	21.2	22.2	-	31.5	20.6	21.5	21,0	20.8	20.8	21.1	21.2	26.2	21.1	28.3	21.1
9 *Komagataeibacter maltaceti*	23.7	23.5	45.5	24.5	31.3	24.2	25.6	31.5	-	22.7	23.5	22.9	22,0	21.6	23.7	23.8	29.6	22.8	29.2	21.8
10 *Komagataeibacter medellinensis*	24.5	25.4	21.1	26.3	20.7	26,0	26.5	20.6	22.7	-	25.3	25.8	32.5	24.3	25.1	26.1	20.8	25.1	21.3	26.6
11 *Komagataeibacter nataicola*	26.5	45.4	23,0	28,0	21.5	28.1	28.3	21.5	23.5	25.3	-	27.1	26.5	24.9	48.9	28.4	23.8	45.4	21.2	26.5
12 *Komagataeibacter oboediens*	27.1	27.2	23.7	40.6	21.1	57.7	28.8	21,0	22.9	25.8	27.1	-	27.5	26.1	27,0	37.3	24.1	27.9	21.6	37.3
13 *Komagataeibacter rhaeticus*	26,0	27.4	21.9	28.2	20.9	27.6	28,0	20.8	22,0	32.5	26.5	27.5	-	25.5	26.7	27.9	21.7	27.1	21.8	27.4
14 *Komagataeibacter saccharivorans*	26.7	25.2	21.7	26.6	21.3	27.2	28.8	20.8	21.6	24.3	24.9	26.1	25.5	-	24.9	26.2	21.6	25.2	21.6	27.2
15 *Komagataeibacter sucrofermentans*	26.9	46.9	22.7	28.1	21.1	28,0	28.3	21.1	23.7	25.1	48.9	27,0	26.7	24.9	-	28.3	23.7	46.6	22,0	26.4
16 *Komagataeibacter swingsii*	28.2	28.3	23.5	46.1	21,0	38.9	29.4	21.2	23.8	26.1	28.4	37.3	27.9	26.2	28.3	-	24.7	28.6	21.2	47,0
17 *Komagataeibacter cocois*	23.8	24,0	29.1	26.1	26.4	25.3	25.7	26.2	29.6	20.8	23.8	24.1	21.7	21.6	23.7	24.7	-	24.8	62.3	21.6
18 *Komagataeibacter xylinus*	26.7	47.3	22.6	29.3	21.4	28.3	27.9	21.1	22.8	25.1	45.4	27.9	27.1	25.2	46.6	28.6	24.8	-	22.2	27,0
19 *Komagataeibacter pomaceti*	21.5	22.2	29.1	22.3	28.4	21.5	22.5	28.3	29.2	21.3	21.2	21.6	21.8	21.6	22,0	21.2	62.3	22.2	-	21.7
20 *Komagataeibacter diospyri*	27.3	21.8	21.6	43.7	21.2	37.8	29.1	21.1	21.8	26.6	26.5	37.3	27.4	27.2	26.4	47,0	21.6	27,0	21.7	-

Supplementary Table 3: List of putative efflux protein subunits in genomes of strains AV382 and AV436.

Type of putative transporter	Strain	
	AV436	AV382
Efflux RND transporter permease subunit		WP_116701804
		WP_116701963
	WP_172154261	WP_116702069
	WP_172154930	WP_116702137
	WP_172155105	WP_116702281
	WP_172156463	WP_116702311
	WP_172156575	WP_116702312
	WP_172157792	WP_116703193
	WP_172157794	WP_116703311
	WP_172158299	WP_116703400
	NPC66221	WP_116703590
		WP_148707809
		WP_148707839
CusA/CzcA family heavy metal efflux RND transporter	WP_172154581	
	WP_172155688	
Multidrug efflux SMR transporter	WP_172155667	WP_116702187
DHA2 family efflux MFS transporter permease subunit	WP_172154795	
	WP_172156362	WP_116702774
	WP_172157129	WP_116702786
	WP_172157284	WP_116702847
	WP_172158620	WP_116703365
	WP_172158636	WP_116703523
	WP_172158899	WP_116704004
	NPC66688	
